# Development of a non-invasive heart rate measurement method for sea turtles with dense keratinous scutes through effective electrode placement

**DOI:** 10.3389/fphys.2024.1511443

**Published:** 2025-01-14

**Authors:** Ayaka Saito, Kino Sakai, Megumi Kawai, Lyu Lyu, Kazunari Kameda, Hiromi Kudo, Katsufumi Sato, Kentaro Q. Sakamoto

**Affiliations:** ^1^ Atmosphere and Ocean Research Institute, The University of Tokyo, Kashiwa, Japan; ^2^ Kuroshima Research Station, Sea Turtle Association of Japan, Taketomi, Japan; ^3^ Center for Research and Education of Wildlife, Kyoto City Zoo, Kyoto, Japan

**Keywords:** heart rate, sea turtle, non-in vasive method, biologging, electrocardiogram

## Abstract

Measuring the heart rate of sea turtles is important for understanding their physiological adaptations to the environment. Non-invasive methods to measure the electrocardiogram (ECG) of sea turtles have been developed by attaching electrodes to their carapace. However, this method has only been applicable to sea turtles with sparse keratin on their shell surfaces, such as loggerhead turtles, and it is difficult to detect heartbeats in sea turtles with dense keratinous scutes, including green sea turtles. Here, we explored the electrode placements on the plastron that can be applied to ECG measurement in green turtles. ECG signals were checked using a handheld ECG monitor at three sets of electrode placement on the plastron. When ECG signals could be detected, they were measured in the water tanks for several days to confirm the clarity of the ECG signals. Of the 29 green turtles, when the negative electrode was placed near the neck area of the plastron, clear ECG signals were obtained in nine individuals (39.1%), whereas ECG signals were not detected at any placements in four individuals (17.4%). Furthermore, in the water tank experiments, continuous ECG signals were successfully recorded by attaching a negative electrode near the neck: almost noiseless clear ECG signals even during moving in seven out of ten individuals and slightly weak and noisy signals in other individuals. The measured heart rate of ten individuals during resting was 8.6 ± 2.9 (means ± s.d.) beats min^−1^ and that during moving was 12.2 ± 4.7 beats min^−1^, similar to those reported in a previous study involving the insertion of electrodes inside the body. Therefore, for measuring the ECG of green turtles, the negative electrode should be placed closer to the neck, and the positive and earth electrodes should be placed to the lower left of the plastron. Although the selection of suitable individuals for measurements is required, this heart rate measurement method will contribute to a better understanding of the physiological status of sea turtles with dense keratinous scutes, including green turtles.

## 1 Introduction

Sea turtles are diving reptiles specialized in the marine environments. Despite being air-breathing animals, they have outstanding abilities for deep (e.g., 1,186 m in leatherback turtles, López-Mendilaharsu et al., 2009; 340 m in loggerhead turtles, Narazaki et al., 2015) and long dives (e.g., 614 min in loggerhead turtles, Broderick et al., 2007; 330 min in green turtles, Fukuoka et al., 2015) among marine reptiles. Moreover, they spend most of their life at sea, except for nesting and hatching on land (Lutcavage et al., 1987; Hochscheid et al., 2010). To understand the physiological adaptations of sea turtles to the marine environment, many studies have been conducted to measure their cardiovascular adjustments using an electrocardiogram (ECG). From the ECG records of free-ranging animals, the number of heartbeats per minute, i.e., the heart rate, can be calculated, which reflects the cardiovascular status at that time. As sea turtles have hard shells, it is difficult to measure ECG by attaching electrodes to their body surfaces, as in terrestrial animals. Therefore, ECG measurements in turtles have been conducted by inserting electrodes inside the body (Southwood et al., 1999, 2003; Williams et al., 2019; Okuyama et al., 2020). Recently, an ECG measurement method has been developed by attaching electrode patches to the surface of the carapace in several species of hard-shelled sea turtles (Sakamoto et al., 2021). However, these non-invasive methods have only been applicable in loggerhead (*Caretta caretta*), black (black morphotypes of the eastern Pacific *Chelonia mydas*; Álvarez-Varas et al., 2021), and olive ridley turtles (*Lepidochelys olivacea*). In some species, such as green (*C. mydas*) and hawksbill turtles (*Eretmochelys imbricata*), ECG signals were hardly detected even when the electrodes were attached to the same positions reported for loggerhead turtles on the carapace. Sakamoto et al. (2021) suggested that the lack of detection may be due to the dense keratinous scutes of these species, making it difficult to transmit biopotentials.

Although non-invasive heart rate measurement methods have been difficult to apply to sea turtle species with dense keratinous scutes, there has been great interest in studying their physiological status. Green turtles, for example, are distributed from temperate to tropical areas and are listed as “Endangered” on the International Union for Conservation of Nature and Natural Resources Red List (Seminoff, 2023). However, conflicts with fishermen have become problematic because of bycatch in set nets and the modification of coastal marine ecosystems associated with seaweed feeding. Heart rate is regulated according to diving behavior under natural environments (Ponganis, 2015). Cardiovascular adjustment including such heart rate regulation is related to the use of oxygen store and is essential for diving ability (Ponganis, 2015). A more sophisticated understanding of sea turtle physiology based on heart rate may provide key insights into solving the issues in sea turtles. Therefore, it is desirable to develop a non-invasive ECG measurement method for sea turtles with dense keratinous scutes. In loggerhead turtles, clear ECG waves could be obtained by changing the electrode placements from the carapace to the plastron, closer to the heart (Kinoshita et al., 2022a). Therefore, we tested whether ECG signals could be obtained by placing the electrodes in different placements from those used in previous studies.

In this study, we explored the effective electrode placement for ECG measurement in green turtles by attaching electrodes to their plastron. First, the quality of the ECG signals obtained from three different electrode placements was evaluated using a handheld ECG monitor. Next, when ECG signals were confirmed, animal-borne recorders were used to check whether ECG signals could be measured continuously for several days in the water tanks. We report that a high percentage of clear ECG signals can be obtained by placing the electrodes closer to the necks of green turtles.

## 2 Materials and Equipment

### 2.1 Animals and study sites

Juvenile green turtles, *C. mydas*, were collected from two regions: the Sanriku coastal area and Kuroshima Island, Japan (Table 1). The Sanriku coastal area of Japan is located in the temperate region of the western North Pacific and is a summer-restricted foraging ground for green turtles (Fukuoka et al., 2015). Fifteen turtles were incidentally captured by commercial set nets between June and August of 2020–2023. Once safely rescued, the turtles were promptly transferred to the marine station of the University of Tokyo (Otsuchi Coastal Research Center, Atmosphere and Ocean Research Institute, The University of Tokyo; 39°21′05″N, 141°56′04″E). Kuroshima Island, on the Yaeyama Islands, located in a subtropical area in southern Japan, provides a year-round foraging habitat for green turtles (Kameda et al., 2017, 2023). Fourteen turtles were captured using an entanglement net (200 m length × 2 m height, mesh size = 40 cm) in February 2023. The net was set in the inner reef area at two sites, the northeast and south sides of Kuroshima Island, and was carefully checked by snorkelers to avoid the risk of mortality of the turtles. Once a turtle was captured, the turtle was promptly transferred to the Kuroshima Research Station, Sea Turtle Association of Japan (24°14′24.0″N, 123°59′36.4″E).

**TABLE 1:** Summary of data for green sea turtles in 2 study sites.

Study site	No. of turtles	*BM* (kg)	*SCL* (cm)
Kuroshima Island	14	15.7 ± 6.7	48.2 ± 6.2
		(6.4–32.1)	(37.0–61.4)
Sanriku coastal area	15	15.6 ± 7.2	46.7 ± 6.7
		(8.0–35.9)	(39.2–64.6)
Total	29	15.6 ± 6.8	47.4 ± 6.4
		(6.4–35.9)	(37.0–64.6)

*BM*, body mass; *SCL*, straight carapace length. Means are reported ± s.d. Numbers in parentheses indicate maximum and minimum values.

After the turtles were transferred to each marine station, the straight carapace length (*SCL*) and body mass (*BM*) were measured. The *BM* and *SCL* of the turtles were 15.6 ± 6.8 (means ± s.d.) kg and 47.4 ± 6.4 cm, respectively (Table 1). The sex of all turtles was not determined. After morphological measurements, they were kept in outdoor flow-through seawater tanks for 1—2 months at most. After the experiments, all the turtles were released into the sea around where they were caught. This study was conducted as a part of a “tag and release” program. All turtles were tagged with metal or plastic ID tags. All experimental procedures were approved by the Animal Ethics Committee of the Atmosphere and Ocean Research Institute at the University of Tokyo (approval numbers P20-11, P21-13, P22-12, P22-21, and P23-21). The study in Kuroshima Island was approved by the Marine Fisheries Coordinating Committee of Okinawa Prefecture, Japan (approval numbers: Oki-cho K4-16).

### 2.2 Electrocardiogram and behavioral recorders

The experiment involved two steps. Firstly, a handheld ECG monitor (Checkme ECG; San-ei Medisys, Kyoto, Japan) was used to confirm the ECG signals for a short period on land. Secondly, if ECG signals were detected by the monitor, animal-borne ECG recorders were used to verify whether ECG signals could be measured for up to 4 days in water tanks. ECG was recorded at 250 Hz using an ECG recorder (ECG400-DT; Little Leonardo, Tokyo, Japan; cuboid shape: 21 mm wide, 64 mm long, 23 mm high, 60 g mass in air; ECG400-D3GT; Little Leonardo; cuboid shape: 31 mm wide, 67 mm long, 17 mm high, 61 g mass in air). Turtle behavior was recorded using an accelerometer (M190L-D2GT; Little Leonardo; cylindrical shape; 15 mm diameter, 53 mm length, 18 g mass in air). The accelerometer and ECG recorder were attached to the turtles along the longitudinal axis of their carapaces. The longitudinal acceleration was recorded at 16 Hz or 50 Hz, and temperature, and depth were recorded at 1 Hz, and 1 Hz, respectively.

## 3 Methods

### 3.1 Confirmation of ECG signals in a short time

**FIGURE 1:**
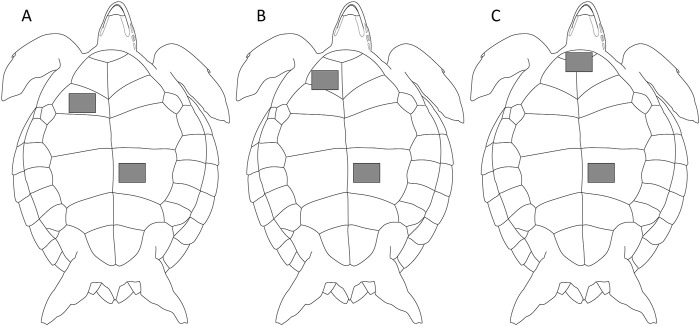
Three positions of electrodes of green sea turtles. Two electrodes were attached to the plastron; the negative electrode was attached to **(A)** humeral scute, the same position reported for loggerhead turtles (Kinoshita et al., 2022a), **(B)** gular scute, or **(C)** intergular scute, and positive and earth electrodes were attached to abdominal scute.

The R wave (depolarization of the ventricles) shows the most drastic change in voltage among the ECG signals and is used to detect heartbeats (Sakamoto et al., 2021). To measure clear R waves, it is suitable to attach the negative electrode to the upper right and both the positive and earth electrodes to the lower left of the plastron for loggerhead turtles (Kinoshita et al., 2022a; Figure 1A). Therefore, these electrodes were attached to different placements of the plastron of green turtles, and the amplitudes and stability of the ECG signals were confirmed by using the handheld monitor. The signals were confirmed on land after the turtles were removed from the tank. For conformation, we used 15 turtles from the Sanriku coastal area and 14 turtles from Kuroshima Island.

Three positions were tested for the negative electrode: humeral scute (Position A; Figure 1A), the same position reported for loggerhead turtles (Kinoshita et al., 2022a), gular scute (Position B; Figure 1B), and intergular scute (Position C; Figure 1C). Positive and earth electrodes were attached to the abdominal scute. Preliminary experiments showed that the detectability of the signal changed depending on the position of the negative electrode, and ECG signals were more likely to be confirmed when the electrode was placed close to the neck. Therefore, we selected Position C, the conventional position (Position A), and Position B, which is in between the two, as the position of the negative electrode. A conductive adhesive tape (KNZ-ST50 shield cloth tape; Kyowa Harmonet Ltd., Kyoto, Japan) was cut into squares (1 cm × 2.5 cm) to make the small electrodes. Small electrodes were attached at each position and connected to a handheld ECG monitor by clipping the electrodes with alligator clips. The handheld ECG monitor displayed ECG signals in real-time. We checked whether the signals could be detected for 30 s to several minutes at each position.

### 3.2 Confirmation of ECG signals in a long time

ECG data may not be applicable for analysis if the ECG contains noise produced by muscle contractions, particularly when the turtle is moving. To confirm whether a qualified ECG could be measured continuously for up to 4 days, animal-borne recorders were attached to 14 turtles (six in the Sanriku coastal area and eight on Kuroshima Island) after confirming successful detection from the handheld ECG monitor.

The ECG signals of the turtles were recorded using a previously described non-invasive method that involved attaching two-electrode patches to the carapace of the turtles (Sakamoto et al., 2021) with some modifications (Kinoshita et al., 2022a). The ECG electrodes were made by cutting conductive adhesive tape into squares (4 cm × 5 cm). A lead wire was attached to the ends of the electrodes using the same adhesive tape. Two electrodes were attached to the plastron where the ECG signals were confirmed by using the handheld monitor in each turtle. In the water tank experiments, the specific electrode placements for each individual were as follows: G2015 at Position A; G2262, G2263, and G2268 at Position B; and other individuals at Position C. After attaching the electrodes, a conductive cream (ECG cream; Suzuken, Aichi, Japan) was applied to the surfaces to allow the electrical signals to pass. As seawater intrusion into the electrodes causes electrical noise in the ECG, the water droplets around the electrodes were carefully removed using acetone. Thereafter, the electrodes were waterproofed with waterproof adhesive plasters (BAND-AID Brand Hydroseal XL bandages; Johnson & Johnson, NJ, United States), and the edges of the adhesive plasters were glued using an instant adhesive (Aron Alpha Jelly Extra; Konishi, Osaka, Japan). Waterproof films (FC waterproof free-cut; Hakujuji, Tokyo, Japan) were used to cover the electrodes completely. To ensure that the electrodes were waterproof, they were thinly coated with an epoxy (Bond Quick 5; Konishi). The lead wires of the electrodes were arranged from the plastron along the side of the body to the carapace and fixed with adhesive tape and instant adhesive. These wires were connected to the lead wires extending from the ECG recorder and sealed with a heat-shrinkable tube. Finally, the ECG recorder and accelerometer were placed on a waterproof adhesive plaster pasted on the carapace using an instant adhesive. The attachment of the instruments was completed within approximately 1 h. After the electrodes were attached, the turtles were returned to their tanks (1 m × 2 m × 1 m).

### 3.3 Analysis

The ECG signals were evaluated based on their amplitudes and stability as “undetectable” when they were visually detectable for less than 30% of the measurement period, “unclear” when they were 30%–70%, and “clear” when they were more than 70%. To analyze ECG, longitudinal acceleration, and temperature data obtained by animal-borne recorders, IGOR Pro version 8.04 (Wavemetrics, Portland, OR, United States) was used with the Ethographer program package (Sakamoto et al., 2009). Data within a 12-h period after returning to the water tank was excluded to eliminate handling effects from the instrument attachments. In the case of turtle G2356, only the ECG and longitudinal acceleration data were analyzed because the temperature data were lost owing to a device error. Small R waves may be partially offset by the noise produced by muscle contractions when the turtle is moving, preventing the accurate detection of R waves. Therefore, the behavior of the turtles was classified into resting or moving phases based on longitudinal acceleration data to calculate the heart rate during each phase (Kinoshita et al., 2022a). The degree of activity of sea turtles can be reflected by the values of the standard deviation of longitudinal acceleration. We defined 0.5 m s^−2^ as the threshold to distinguish between resting and moving phases (sea turtles were hardly moving when the value was below 0.5 m s^−2^). The standard deviation of the longitudinal acceleration was calculated every minute; values lower than 0.5 m s^−2^ were regarded as representing resting status, otherwise as moving status. Only the same status that continued for at least 2 min was defined as the corresponding phase. The ECG data were processed using the ECGtoHR package (Sakamoto et al., 2021), which runs on IGOR Pro. After band-pass filtering, the R waves were detected. For each turtle, the heart rate during each phase (resting and moving) was calculated by dividing the total number of R-waves per period by the duration of each period. These values were averaged to obtain the grand mean value for each phase. The values are expressed as means ± s.d.

## 4 Results

The ECG signals of the 29 green turtles were checked for three sets of electrode placements on the plastron using a handheld ECG monitor (Table 2). By attaching electrodes at Position A, the same position reported for loggerhead turtles, no signal was detected in any individual. On the other hand, clear ECG signals were detected when the negative electrode was attached closer to the neck with signals detected from four individuals (14.3%) at Position B and nine individuals (39.1%) at Position C. When unclear ECG signals were detected at Position A, the same individual showed clear signals by attaching the electrodes at Position C, the placement closest to the neck (nine individuals, 31.0%; Supplementary Table S1). However, no matter where the electrodes were placed, ECG signals were unclear from ten individuals (34.5%) and undetectable from four individuals (17.4%). Among the individuals used in this study, the body size and habitat did not have any obvious effects on the clarity of the ECG signal of ECG measurements (Supplementary Table S1).

**TABLE 2:** Summary of evaluations for 3 sets of electrode placements using handheld ECG monitor.

Evaluation	Electrode placement					
	Position A		Position B		Position C	
Undetectable	4	(16.7%)	4	(14.3%)	4	(17.4%)
Unclear	20	(83.3%)	20	(71.4%)	10	(43.5%)
Clear	0	(0%)	4	(14.3%)	9	(39.1%)
Total	24		28		23	

**FIGURE 2:**
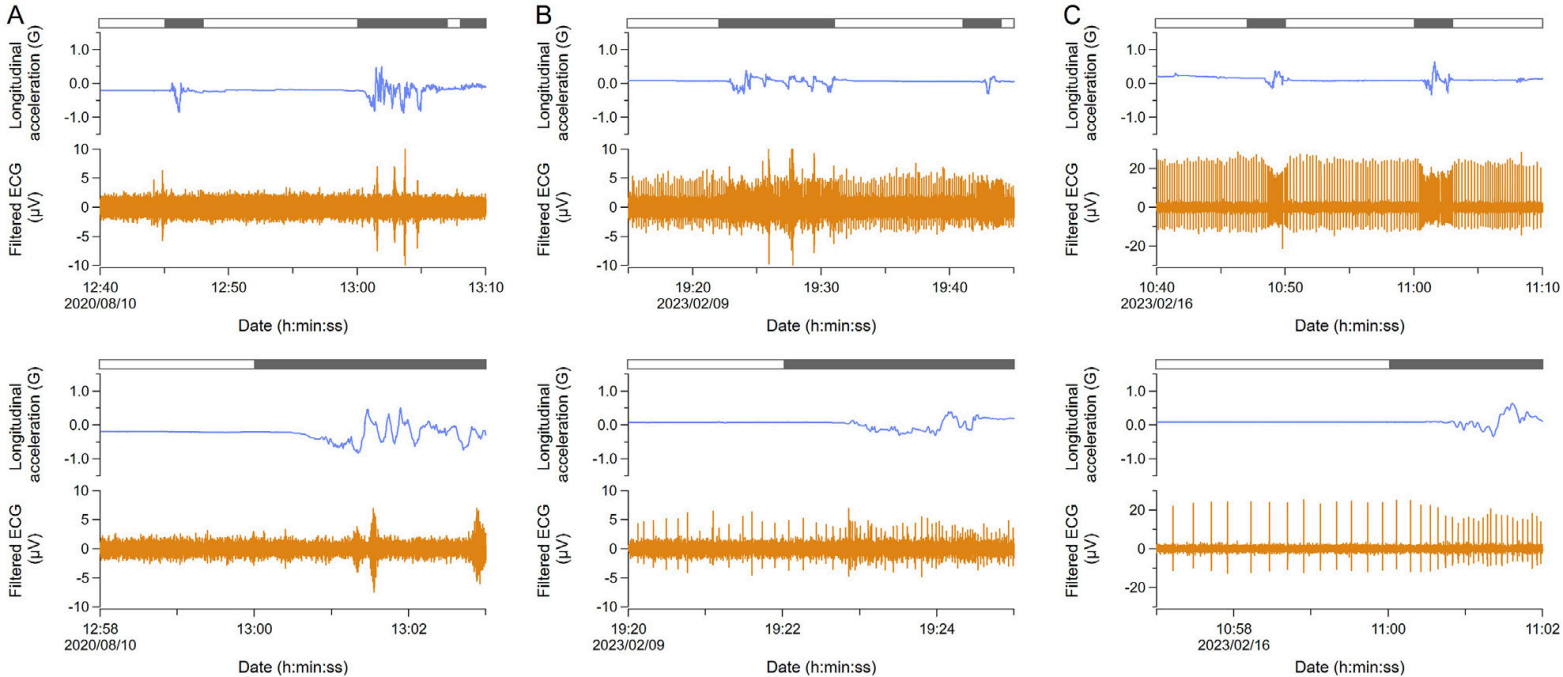
Comparison of ECG signals measured with animal-borne recorders. In each section, the top figure shows the data for 30 min and the bottom one is the enlarged data. Blue and orange lines indicate longitudinal acceleration and filtered ECG, respectively. The bar above indicates moving (black) and resting (white) phases. **(A)** ECG signals were undetectable (G2015; Position A). **(B)** ECG signals were unclear. ECG waves contained some noises produced by muscle contraction, especially during moving (KG2308; Position C). **(C)** ECG signals were clear (KG2310; Position C). Therefore, placing the electrode at position C is recommended. Note that the range of the *Y* axis in ECG is different from others.

After the ECG signals were confirmed using a handheld ECG monitor, the ECG and behavior of 14 turtles were measured for 2–4 days using animal-borne recorders (Supplementary Table S1). In one individual with electrodes placed at Position A, ECG signals were undetectable (Figure 2A). Three individuals with electrodes placed at Position B showed clear ECG signals even during the moving phases, although they were not tested at Position C using a handheld ECG monitor (Supplementary Table S1). For the ten individuals with electrodes placed at Position C, closest to the neck, the signals were continuously confirmed. While three of them showed unclear ECG signals, specifically during the moving phases (Figure 2B), seven of them showed clear ECG signals with almost no noise even during moving phases (Figure 2C). The mean heart rate of ten individuals was 8.6 ± 2.9 beats min^−1^ (n = 2,520) during resting phases and 12.2 ± 4.7 beats min^−1^ (n = 1991) during moving phases. The average water temperature during the experiments was 23.4°C ± 1.1°C (the Sanriku coastal area; 24.3°C ± 0.2°C, Kuroshima Island; 23.3°C ± 1.2°C).

## 5 Discussion

In green turtles, clear ECG signals could be obtained from a higher percentage of individuals by placing the negative electrode closer to the neck on the plastron. Both the positive and earth electrodes were placed to the lower left as shown in Kinoshita et al. (2022a), whereas the negative electrode should be placed close to the neck. The variations in the quality of the signals within individuals may be due to differences in electrical conductivity among the positions of the plastron for some reasons. For example, the scapula (shoulder blade) is under the humeral scute (Wyneken, 2001), and the bones do not transmit electricity. In addition, the border of the scutes near the neck may allow biopotentials to easily transmit. Therefore, factors such as the position of bone, the thickness of the plastron, and the amount of muscle and fat may affect the electro-conductivity of the body. Necropsy data such as the presence of bones and tissues under various scutes and the composition of plastron will guide the optimal electrode placement for future heart rate measurements in sea turtles. Moreover, body size had no obvious effect on the position of the electrodes and the clarity of the ECG signals among the individuals used in this study. However, the effect of larger body size and growth stage have not been confirmed in green turtles. Further study is needed to determine whether body size affects the percentage of individuals available for heart rate measurements. At that time, it will be effective to adjust the electrode placements using a handheld ECG monitor, as in this study.

In a previous study that measured ECG by inserting electrodes inside the body, the heart rate of juvenile green turtles (*BM*; 19.4 ± 1.5 kg, *SCL*; 53.8 ± 1.4 cm) was reported to be 11.1 ± 0.4 beats min^−1^ during resting dives and 15.0 ± 0.5 beats min^−1^ during active dives (Okuyama et al., 2020). The values in this study are slightly lower than those in the previous study, possibly due to the lower water temperature (this study; 23.4°C ± 1.1°C, Okuyama et al., 2020; 29.0°C ± 0.0°C). Therefore, the heart rate measured in this study was similar to that obtained by inserting electrodes into the body, suggesting that this method allowed us to obtain a highly accurate ECG of green turtles. The amplitude of the ECG signal in this study was approximately one-tenth of the signal obtained using the conventional approach; however considering band-pass filter processing, this amplitude is sufficient to detect R waves.

Our method is minimally stressful for sea turtles and can be applied to monitor the health of captive individuals in aquariums. It can also be used to measure the heart rate of sea turtles in the field without recapturing them by using an automated tag release system for removing the electrodes and recorders (Saito et al., 2024). This helps us to understand the physiological status of sea turtles under natural conditions. By using our method, ECG of green turtles could be measured stably for at least 4 days. This was the limit of the battery in the recorder, but the electrodes remained attached to the plastron in the water tank for about a week. In the future, ECG might be measured for longer periods by improving the wires and electrodes. The non-invasive ECG measurement method is now applicable to sea turtles, which was previously unavailable. Diving ability, such as dive duration and depth varies among species; therefore, a comparison of heart rates among sea turtle species may reveal the physiological functions underlying differences in diving ability. Heart rate is used as a proxy for indirectly estimating field metabolic rates on a fine time scale (Kinoshita et al., 2022b). In addition, when sea turtles are entangled in fishing nets, their heart rates will increase due to higher activity levels, potentially causing gas emboli and rapid oxygen depletion (Williams et al., 2019). Studying heart rate of sea turtles during diving and various levels of activity can lead to a better understanding of energetics under natural environments and the physiological effects of interrupting natural diving patterns. Such knowledge will be fundamental to assessing the potential impacts of the issues in sea turtles, including bycatch and the modification of coastal marine ecosystems.

This study successfully identified an effective electrode placement for heart rate measurement in green turtles. The negative electrode should be placed closer to the neck, and both the positive and earth electrodes should be placed to the lower left of the plastron. While the ECG signals in some individuals were less distinct or undetectable regardless of placement, the use of a handheld monitor, as demonstrated in this study, offers a practical approach for selecting suitable individuals, especially for turtles with dense keratinous scutes, such as green turtles. Green and hawksbill turtles, which face extinction, primarily use coastal areas after growing up and are closely related to human activities through bycatch and feeding damage. To coexist with sea turtles, it is important not only to understand their ecology but also to closely examine their physiological status such as their heart rate. Our method contributes to a better understanding of the physiological status of free-ranging sea turtles.

## Data Availability

The original contributions presented in the study are included in the article/Supplementary Material, further inquiries can be directed to the corresponding author.
