# Supplementary material for: Development of a non-invasive heart rate measurement method for sea turtles with dense keratinous scutes through effective electrode placement
Source: Front Physiol. 2025 Jan 14;15:1511443. doi: 10.3389/fphys.2024.1511443 (PMC11772307; doi:10.3389/fphys.2024.1511443)
Supplement: Supplementary file 1 [file Table1.docx]

Supplementary Table 1. Evaluation of ECG signals for all individuals

| ID | Study site | *BM* (kg) | *SCL* (cm) | Confirmation by  a handheld ECG monitor | | | Confirmation by  animal-borne recorders |
| --- | --- | --- | --- | --- | --- | --- | --- |
|  |  |  |  | Position A | Position B | Position C |  |
| G2015 | S | 10.5 | 42.4 | 1 | ‒ | ‒ | 0 |
| G2262 | S | 14.6 | 46.0 | ‒ | 2 | ‒ | 2 |
| G2263 | S | 16.3 | 48.8 | ‒ | 2 | ‒ | 2 |
| G2264 | S | 10.8 | 44.5 | ‒ | 1 | ‒ | ‒ |
| G2268 | S | 12.5 | 44.9 | ‒ | 2 | ‒ | 2 |
| G2269 | S | 13.9 | 43.5 | ‒ | 1 | ‒ | ‒ |
| G2356 | S | 8.0 | 39.2 | 1 | 1 | 2 | 2 |
| G2361 | S | 10.2 | 40.5 | 1 | 2 | 2 | ‒ |
| G2362 | S | 12.8 | 44.1 | 1 | 1 | 2 | ‒ |
| G2371 | S | 15.3 | 45.6 | 1 | 1 | 1 | ‒ |
| G2392 | S | 22.0 | 52.0 | 1 | 1 | 2 | ‒ |
| G2394 | S | 25.4 | 57.3 | 1 | 1 | 1 | 1 |
| G2395 | S | 35.9 | 64.6 | 1 | 1 | 2 | ‒ |
| G2396 | S | 11.2 | 42.0 | 1 | 1 | 1 | ‒ |
| G2399 | S | 14.4 | 45.3 | 1 | 1 | 1 | ‒ |
| KG2302 | K | 17.7 | 50.0 | 1 | 1 | 1 | ‒ |
| KG2303 | K | 13.2 | 47.0 | 0 | 0 | 0 | ‒ |
| KG2304 | K | 12.3 | 45.6 | 0 | 0 | 0 | ‒ |
| KG2305 | K | 8.4 | 41.1 | 1 | 1 | 1 | 1 |
| KG2306 | K | 6.4 | 37.0 | 0 | 0 | 0 | ‒ |
| KG2308 | K | 15.7 | 47.1 | 1 | 1 | 2 | 1 |
| KG2309 | K | 23.5 | 55.7 | 1 | 1 | 1 | 2 |
| KG2310 | K | 32.1 | 61.4 | 1 | 1 | 2 | 2 |
| KG2311 | K | 11.0 | 43.9 | 1 | 1 | 1 | 2 |
| KG2312 | K | 18.5 | 50.7 | 1 | 1 | 1 | ‒ |
| KG2313 | K | 10.9 | 44.8 | 1 | 1 | 2 | 2 |
| KG2314 | K | 13.4 | 48.3 | 1 | 1 | 2 | 2 |
| KG2315 | K | 14.9 | 46.9 | 1 | 1 | 1 | 2 |
| KG2316 | K | 21.2 | 55.3 | 0 | 0 | 0 | ‒ |

S: Sanriku coastal area; K: Kuroshima Island; *BM*: body mass; *SCL*: straight carapace length; 0: Undetectable; 1: Unclear; 2: Clear; ‒: No data. Position A, B, and C indicate electrode placements (Fig. 1).
